# Growth differentiation factor 6, a repressive target of EZH2, promotes the commitment of human embryonic stem cells to mesenchymal stem cells

**DOI:** 10.1038/s41413-020-00116-y

**Published:** 2020-11-17

**Authors:** Pend Deng, Yongxin Yu, Christine Hong, Cun-Yu Wang

**Affiliations:** 1 Laboratory of Molecular Signaling, Division of Oral Biology and Medicine, School of Dentistry, University of California Los Angeles, Los Angeles, CA 90095 USA; 2 Section of Orthodontics, Division of Growth and Development, School of Dentistry, University of California Los Angeles, Los Angeles, CA 90095 USA; 3 Jonsson Comprehensive Cancer Center, Broad Stem Cell Research Institute and Department of Bioengineering, Henry Samueli School of Engineering and Applied Science, University of California Los Angeles, Los Angeles, CA 90095 USA

**Keywords:** Bone, Pathogenesis

## Abstract

Mesenchymal stem cells (MSCs) derived from human embryonic stem cells (hESCs) have significant potential for cell-mediated bone regeneration. Our recent study revealed that inhibiting the epigenetic regulator EZH2 plays a key role in promoting the mesodermal differentiation of hESCs. In this study, an epigenome-wide analysis of hESCs and MSCs revealed that growth differentiation factor 6 (GDF6), which is involved in bone formation, was the most upregulated gene associated with MSCs compared to hESCs. Furthermore, we identified GDF6 as a repressive target of EZH2 and found that ectopic GDF6 selectively promoted hESC differentiation towards the mesodermal lineage and enriched the MSC population. Our results provide molecular insights governing the mesenchymal commitment of hESCs and identify an inducing factor that offers strong promise for the future of regenerative medicine.

## Introduction

Mesenchymal stem cells (MSCs) are a promising source for the treatment of various bone diseases and injuries due to their self-renewal capacity and multilineage differentiation potential.^1–3^ MSCs have been isolated from various fetal and adult tissues, including umbilical cord, blood, bone marrow, adipose tissue, and craniofacial tissues.^4–7^ In particular, MSCs derived from embryonic stem cells (ES-MSCs) are an attractive source for cell-based therapies because they exhibit high proliferation and osteogenic potential.^8,9^ However, while there has been meaningful progress in developing protocols to derive MSCs from human embryonic stem cells (hESCs),^10–12^ selectively promoting hESCs to commit to ES-MSCs remains a major challenge. Identifying potential factors that can shift hESCs to differentiate into a mesodermal lineage will lead to advancements in regenerative medicine and facilitate understanding of the early stages of human development. Cell fate determination of hESCs requires intricate coordination between genetic and epigenetic programming.^13^ In hESCs, a high density of repressive epigenetic signatures, such as H3K27me3, silences a large group of developmental regulators that are involved in cell differentiation.^14–16^ Upon developmental cues, these repressive epigenetic profiles are selectively eliminated, thus allowing activation of these genes and subsequent differentiation of cells.^14,15^

Our group recently identified an epigenetic mechanism that promotes the differentiation of hESCs into MSCs.^16^ We demonstrated that preventing enhancer of zeste homolog 2 (EZH2) from inserting the repressive H3K27me3 epigenetic signature enriches hESC differentiation towards MSCs. However, the downstream targets of EZH2 remain largely unknown. Identifying these targets might help to develop a new strategy to generate MSCs from hESCs more efficiently for regenerative medicine and to better understand the molecular mechanism of EZH2-mediated repression. In this study, we compared epigenomic changes relating to histone modifications and the transcriptome between hESCs and MSCs using publicly available RNA sequencing (RNA-seq) and chromatin immunoprecipitation sequencing (ChIP-seq) data. Consistently, this epigenome-wide map revealed that EZH2 was enriched at the promoters of genes belonging to the Wnt and TGF-β signaling superfamily in hESCs, but this enrichment was significantly reduced in MSCs. We discovered that growth differentiation factor 6 (GDF6; also known as BMP13) was the most upregulated gene in MSCs compared to hESCs. GDF6 is a member of the TGF-β superfamily, which is associated with the maintenance and differentiation of ESCs. Interestingly, GDF6 was expressed in the nucleus pulposus of intervertebral discs and hypertrophic chondrocytes during early ossification of vertebrae.^17^ Mutations in *GDF6* are associated with vertebral segmentation defects in Klippel-Feil syndrome and multiple synostoses syndrome.^18,19^ In mice, GDF6 is expressed in frontal bone primordia from embryonic day (E) 10.5 through E12.5 and in a striped pattern across developing skeletal condensations at E13.5.^20,21^ Knocking out *Gdf6* results in defects in the joint, ligament, and cartilage during limb formation and fusion of the coronal suture,^20,21^ indicating that GDF6 plays a critical role in the regulation of mesenchymal differentiation during embryonic development. Therefore, we examined whether GDF6 was a direct target of EZH2 and found that GDF6 potently accelerated the differentiation of hESCs into MSCs with high osteogenic potential, suggesting its strong potential for use in bone regenerative therapies.

## Results

### GDF6 is a target of EZH2 in hESCs and is upregulated during hESC commitment to MSCs

**Fig. 1:**
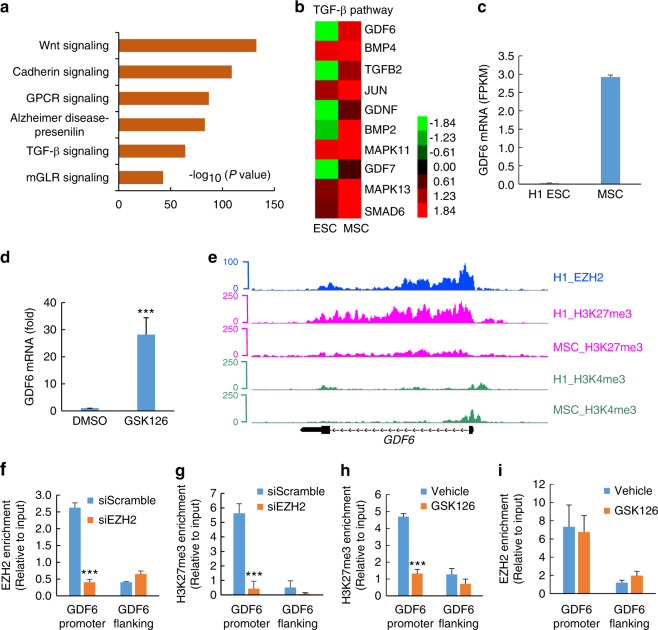
Epigenetic regulation of GDF6 during H1 hESC differentiation towards MSCs. **a** Gene ontology enrichment analysis of genes associated with EZH2 within 3 kb of their TSS as indicated with the GREAT annotation tool. The GO terms include PANTHER pathways. Bars represent −log_10_ of binomial raw *P* values. **b** Heatmap representing the expression of the indicated TGF-β signaling genes enriched for EZH2. **c** GDF6 mRNA was highly expressed in MSCs compared with that in hESCs. **d** qRT-PCR analysis of GDF6 mRNA levels in H1 hESCs treated with DMSO or 10 μmol·L^−1^ GSK126. **e** Genome browser views of EZH2, H3K4me3, and H3K27me3 peaks along the GDF6 gene. For EZH2, enrichment data were only available in H1 hESCs. For histone markers, enrichment data are presented in H1 hESCs and H1 hESC-derived MSCs. **f** EZH2 ChIP-qPCR for GDF6 in siScramble- and siEZH2-transfected H1 hESCs. **g** H3K27me3 ChIP-qPCR for GDF6 in siScramble- and siEZH2-transfected H1 hESCs. **h** H3K27me3 ChIP-qPCR for GDF6 in control and GSK126-treated H1 hESCs. **i** EZH2 ChIP-qPCR for GDF6 in control and GSK126-treated H1 hESCs. All in vitro experiments were performed independently three times. The data are expressed as the means ± SDs. *P < 0.05, **P < 0.01, and ***P < 0.001. For **d**, Student’s *t* test; for **f**–**i**, two-way ANOVA with Tukey’s post hoc test

To determine the pathways associated with EZH2 enrichment, we used the Genomic Regions Enrichment of Annotations Tool (GREAT) and found that EZH2 binds to the promoters of genes associated with the Wnt, Cadherin, and TGF-β signaling pathways in hESCs (*P* < 10^−50^) (Fig. 1a). Within the TGF-β superfamily, GDF6, which is involved in bone formation, was the most upregulated gene among those with EZH2 enrichment (Fig. 1b). Consistently, the expression levels of GDF6 were also higher in MSCs than in hESCs based on RNA-seq analysis (Fig. 1c). We treated H1 hESCs with GSK126, a selective small-molecule-inhibitor of EZH2, and observed that *GDF6* expression was significantly increased by GSK126 as determined by quantitative reverse transcriptase-polymerase chain reaction (qRT-PCR) (Fig. 1d).

To explore the epigenetic regulation of GDF6, we examined the enrichment of H3K4me3 and H3K27me3 in H1 hESCs and MSCs at the GDF6 promoter. Further analysis indicated that H3K27me3 enrichment was significantly lower in MSCs than in hESCs, whereas H3K4me3 enrichment did not exhibit any discernible changes between the two cell types (Fig. 1e), reflecting the reduction in EZH2 activation in MSCs compared to hESCs. To verify whether EZH2 epigenetically regulates GDF6, we used siRNA to knock down EZH2 in H1 hESCs and performed ChIP-qPCR, the results of which revealed that the enrichment of both EZH2 and H3K27me3 at the GDF6 promoter was significantly decreased (Fig. 1f, g). In addition, we treated H1 hESCs with GSK126 and found that the enrichment of H3K27me3 at the GDF6 promoter was significantly decreased while EZH2 binding remained unchanged (Fig. 1h, i). Our findings suggest that H3K27me3 enrichment at the GDF6 promoter is dependent on EZH2 enzymatic activity.

### GDF6 promotes hESC differentiation into MSCs

**Fig. 2:**
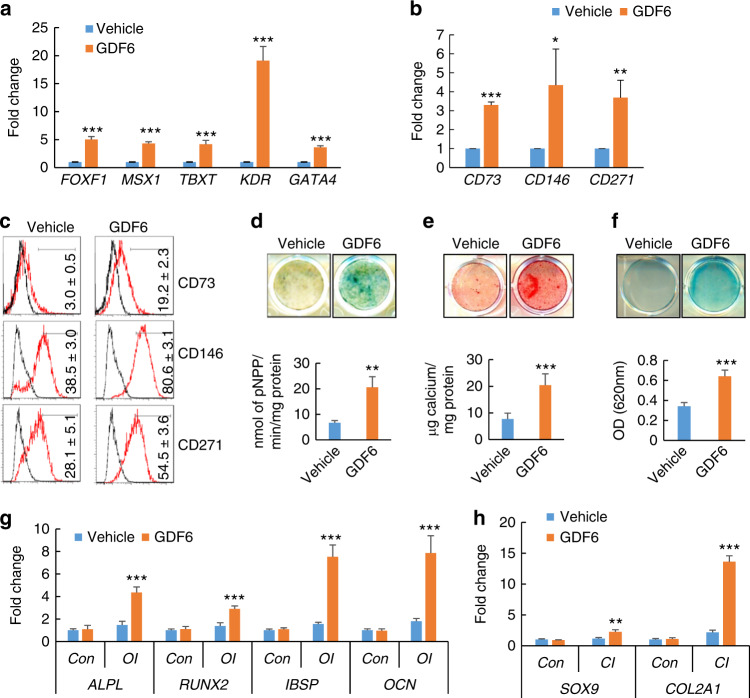
Effect of GDF6 treatment on the mesenchymal lineage commitment of H1 hESCs. **a** qRT-PCR analysis of the expression of mesodermal genes (*FOXF1*, *MSX1*, *TBXT*, *KDR*, *GATA4*) in H1 hESCs treated with 300 ng·mL^−1^ GDF6. **b** qRT-PCR analysis of the expression of MSC surface markers (CD73, CD146, and CD271) in H1 hESCs treated with 300 ng·mL^−1^ GDF6. **c** Flow cytometry analysis of CD73, CD146, and CD271 expression in vehicle- and GDF6-treated H1 cells. **d** ALP staining and quantitative ALP activity assay after 14 days of osteogenic induction (OI) for vehicle- or GDF6-treated H1 cells. **e** ARS staining and quantification after 14 days of OI for vehicle- or GDF6-treated H1 cells. **f** Alcian blue staining and quantification after 21 days of chondrogenic induction (CI) for vehicle- or GDF6-treated H1 cells. qRT-PCR gene expression analysis of osteogenic markers (*ALPL*, *RUNX2*, *IBSP*, *OCN*) (**g**) and chondrogenic markers (*SOX9* and *COL2a1*) (**h**) after lineage-specific differentiation of H1 cells cultured in the presence or absence of GDF6. All in vitro experiments were performed three times independently. *P < 0.05, **P < 0.01, ***P < 0.001. For **a**–**f**, Student’s *t* test; for **g** and **h**, two-way ANOVA with Tukey’s post hoc test

Since GDF6 was a repressive target of EZH2, we assessed whether GDF6 could promote hESC differentiation into MSCs by treating hESCs with GDF6 for 3 days and examining the expression levels of both mesodermal and MSC markers. We found that the mRNA expression of the mesoderm markers *FOXF1*, *MSX1*, *TBXT, KDR*, and *GATA4* was significantly elevated following GDF6 treatment (Fig. 2a). Similarly, gene expression of the MSC markers *CD73*, *CD146*, and *CD271* was also significantly elevated after 5 days of monolayer culture (Fig. 2b). Fluorescence-activated cell sorting (FACS) analysis confirmed that there was a higher yield of CD73^+^CD146^+^CD271^+^ MSCs from H1 hESCs treated with GDF6 (Fig. 2c). Next, we examined whether these MSCs had increased potential for multilineage differentiation. Indeed, MSCs derived from GDF6-treated H1 hESCs exhibited increased ALP activity (Fig. 2d) and mineralization capacity (Fig. 2e) following osteogenic induction (OI). They also displayed enhanced chondrogenesis as indicated by Alcian blue staining upon chondrogenic induction (Fig. 2f). qRT-PCR confirmed enhanced osteogenic potential MSCs derived from GDF6-treated H1 hESCs based on the significant increase in the expression levels of the osteogenic markers *ALPL*, *RUNX2*, *IBSP*, and *OCN* (Fig. 2g). Similarly, the expression levels of the chondrogenic markers *SOX9* and *COL2A1* were also significantly elevated in these cells upon chondrogenic induction (Fig. 2h).

**Fig. 3:**
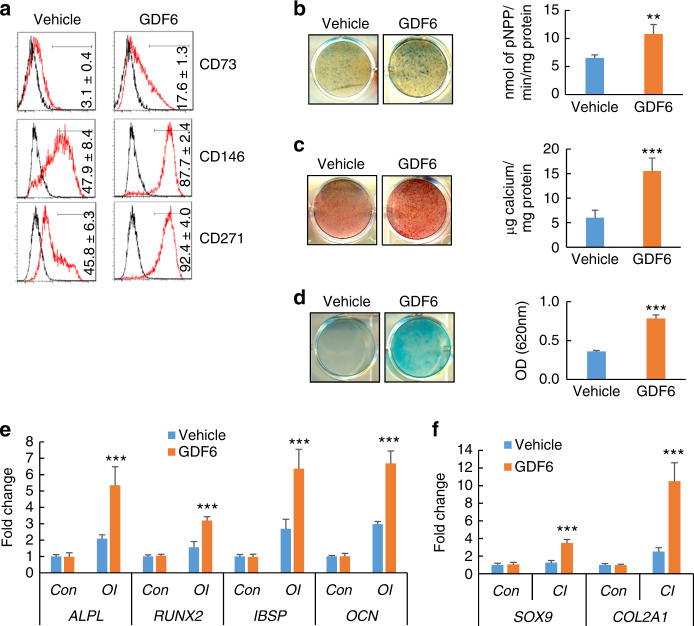
Effect of GDF6 treatment on mesenchymal lineage commitment of H9 hESCs. **a** Flow cytometry analysis of CD73, CD146, and CD271 expression in vehicle- and GDF6-treated H9 cells. **b** ALP staining and quantitative ALP activity assay after 14 days of OI for vehicle- and GDF6-treated H9 cells. **c** ARS staining and quantification after 14 days of OI for vehicle- or GDF6-treated H9 cells. **d** Alcian blue staining and quantification after 21 days of CI for vehicle- or GDF6-treated H9 cells. qRT-PCR gene expression analysis of osteogenic (*ALPL*, *RUNX2*, *IBSP*, *OCN*) (**e**) and chondrogenic markers (*SOX9* and *COL2a1*) (**f**) after lineage-specific differentiation in H9 cells cultured in the presence or absence of GDF6. All in vitro experiments were performed three times independently. **P < 0.01, ***P < 0.001. For **b**–**d**, Student’s *t* test; for **e** and **f**, two-way ANOVA with Tukey’s post hoc test

To confirm these results, we studied whether GDF6 treatment elicited a similar pattern in H9 hESCs. As shown in Fig. 3a, the expression of MSC markers, including CD73, CD146, and CD271, was significantly elevated in the GDF6-treated group compared to the control group. They exhibited enhanced osteogenic potential as revealed by ALP staining and Alizarin Red S (ARS) staining (Fig. 3b, c). The chondrogenic potential of the cells derived from GDF6-treated H9 hESCs was also enhanced (Fig. 3d). Consistent with these data, the expression levels of osteogenic and chondrogenic markers were significantly increased in GDF6-treated H9 hESCs (Fig. 3e, f). Collectively, our data suggest that the TGF-β superfamily member GDF6 enhances the differentiation of hESCs into the mesodermal lineage, resulting in an increased ES-MSC population with osteogenic and chondrogenic differentiation potential.

### Purified MSCs derived from GDF6-treated hESCs exhibit multipotency in vitro and can generate ectopic bone in vivo

**Fig. 4:**
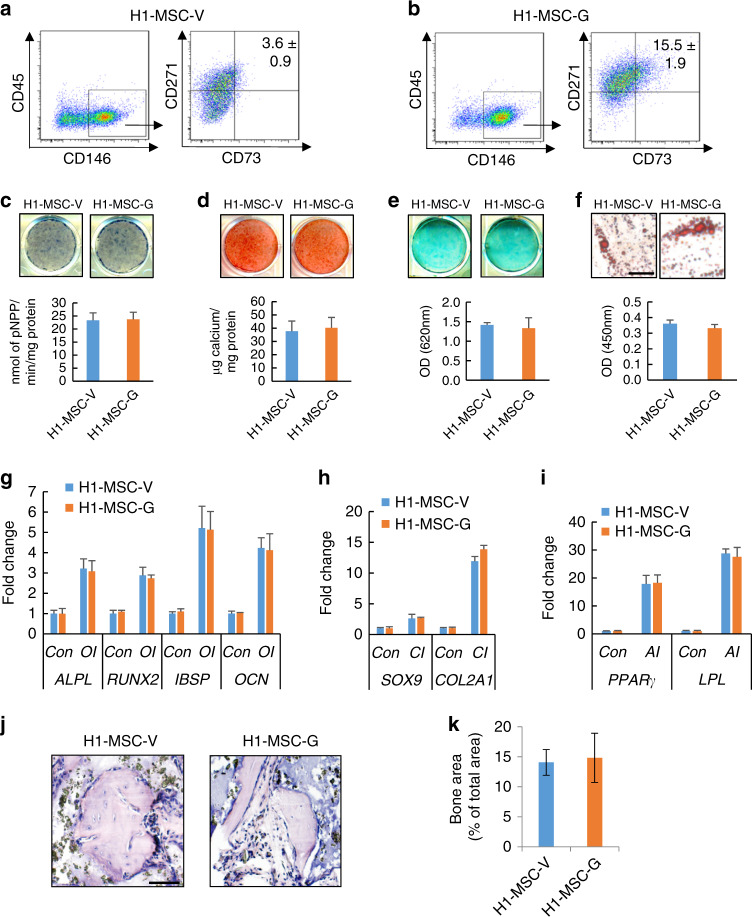
Characterization of purified ES-MSCs derived from GDF6-treated H1 hESCs. Proportions of CD73^+^CD146^+^CD271^+^CD45^−^ H1-MSC-V (**a**) and H1-MSC-G (**b**) were compared by FACS. **c** ALP staining and quantitative ALP activity assay in H1-MSC-V and H1-MSC-G cells after 14 days of OI. **d** ARS staining and quantification in H1-MSC-V and H1-MSC-G cells after 14 days of OI. **e** Alcian blue staining and quantification in H1-MSC-V and H1-MSC-G cells after 21 days of CI. **f** Oil Red O staining and quantification after 21 days of adipogenic induction (AI) in H1-MSC-V and H1-MSC-G cells. Bar indicates 40 μm. qRT-PCR gene expression analysis of osteogenic (*ALPL*, *RUNX2*, *IBSP*, *OCN*) (**g**), chondrogenic (*SOX9* and *COL2a1*) (**h**), and adipogenic markers (*PPARγ* and *LPL*) (**i**) after lineage-specific differentiation in H1-MSC-V and H1-MSC-G cells. **j**, **k** In vivo bone formation of H1-MSC-V and H1-MSC-G cells in immunocompromised mice. Representative H&E staining of transplants (**j**) and quantitative analysis of mineralized tissue versus total area (**k**). *n* = 5. Bar indicates 40 μm. All in vitro experiments were performed three times independently. *P < 0.05, **P < 0.01, ***P < 0.001. For **c**–**f** and **k**, Student’s *t* test; for **g**–**i**, two-way ANOVA with Tukey’s post hoc test

Since our prior experiments showed that inhibiting EZH2 in hESCs resulted in an increased pool of ES-MSCs without affecting their terminal differentiation rate,^16^ we sought to determine whether ectopic GDF6 would elicit a similar effect. First, we demonstrated that treatment with GDF6 (H1-MSC-G) resulted in a fourfold increase in CD73^+^CD146^+^CD271^+^CD45^−^ MSCs compared to that of cells treated with a vehicle control (H1-MSC-V; Fig. 4a, b). Since GDF6 treatment generated more MSCs, it was important to determine whether GDF6-induced MSCs retained their differential potential. Therefore, we compared the osteogenic, chondrogenic, and adipogenic differentiation potentials of CD73^+^CD146^+^CD271^+^CD45^−^ MSCs from GDF-treated H1 hESCs with those from vehicle-treated H1 hESCs. We found no discernible difference in the osteogenic (Fig. 4c, d), chondrogenic (Fig. 4e), or adipogenic (Fig. 4f) differentiation capacity between H1-MSC-G and H1-MSC-V cells. In addition, qRT-PCR showed unchanged expression levels of osteogenic (Fig. 4g), chondrogenic (Fig. 4h) and adipogenic markers (Fig. 4i) between H1-MSC-G and H1-MSC-V cells. Importantly, when extrapolated to an in vivo setting, we found that H1-MSC-V and H1-MSC-G were both able to form bone tissue (Fig. 4j, k).

**Fig. 5:**
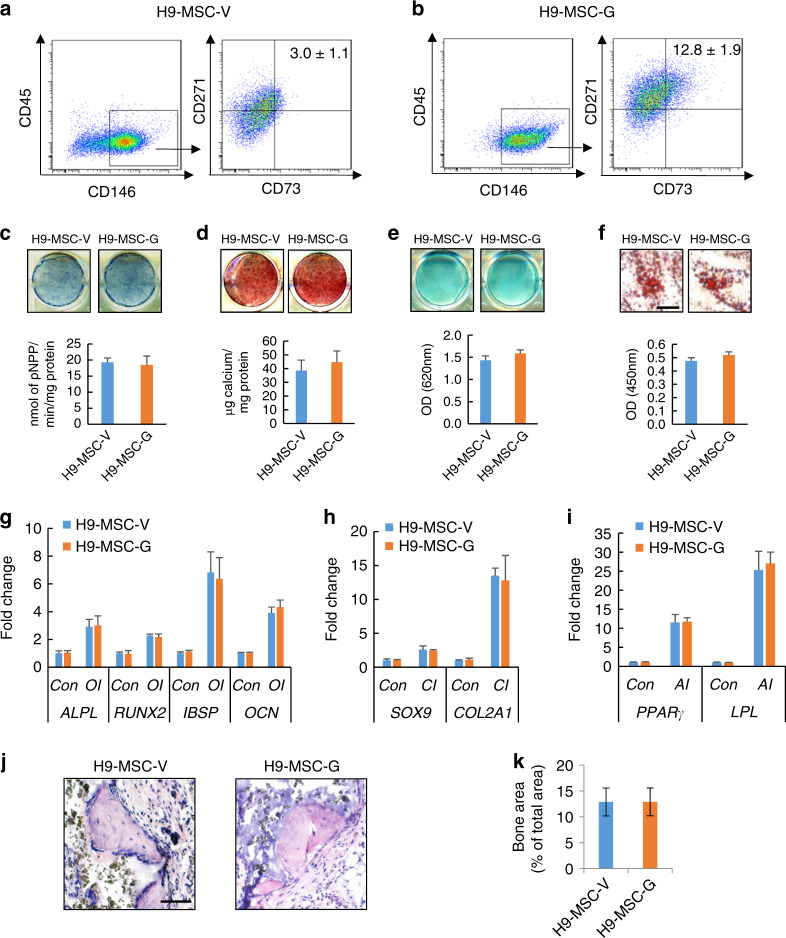
Characterization of purified ES-MSCs derived from GDF6-treated H9 hESCs. The proportions of CD73^+^CD146^+^CD271^+^CD45^–^ H9-MSC-V (**a**) and H9-MSC-G (**b**) cells were compared. **c** ALP staining and quantitative ALP activity assay in H9-MSC-V and H9-MSC-G cells after 14 days of OI. **d** ARS staining and quantification in H9-MSC-V and H9-MSC-G cells after 14 days of OI. **e** Alcian blue staining and quantification in H9-MSC-V and H9-MSC-G cells after 21 days of CI. **f** Oil Red O staining and quantification in H9-MSC-V and H9-MSC-G cells after 21 days of AI. Bar indicates 40 μm. qRT-PCR gene expression analysis of osteogenic (*ALPL*, *RUNX2*, *IBSP, OCN*) (**g**), chondrogenic (*SOX9* and *COL2a1*) (**h**), and adipogenic markers (*PPARγ* and *LPL*) (**i**) after lineage-specific differentiation of H9-MSC-V and H9-MSC-G cells. **j**, **k** Bone formation in vivo by H9-MSC-V and H9-MSC-G in immunocompromised mice. Representative H&E staining of transplants (**j**) and quantitative analysis of mineralized tissue versus total area (**k**) are shown. *n* = 5. Bar indicates 40 μm. All in vitro experiments were performed three times independently. *P < 0.05, **P < 0.01, ***P < 0.001. For **c**–**f** and **k**, Student’s *t* test; for **g**–**i**, two-way ANOVA with Tukey’s post hoc test

Additionally, we observed that compared with the vehicle control, GDF6 treatment significantly generated more MSCs from H9 hESCs (Fig. 5a, b). Similarly, MSCs derived from H9 hESCs (H9-MSC-G) also retained osteogenic, chondrogenic, and adipogenic differentiation potential (Fig. 5c–i). In vivo transplantation demonstrated that both H9-MSC-V and H9-MSC-G were capable of generating bone tissues (Fig. 5j, k). Altogether, our data suggest that exogenous addition of GDF6 to hESCs promotes increased differentiation towards the MSC lineage.

## Discussion

The first discovery of embryonic pluripotency in vitro transformed the field of stem cell biology. Decades later, there are vast current and future clinical applications for hESCs, with ongoing trials showing promising efficacy. However, due to the potential risk of teratoma formation from in vivo regenerative therapy with ESCs,^10,22^ MSCs have gained increasing popularity for bone and cartilage regeneration. In this study, we identified GDF6 as a repressive target of EZH2 that facilitated hESC differentiation into the mesodermal lineage and subsequently generated a greater number of MSCs with high osteogenic potential.

Genome-wide analysis of hESCs revealed that EZH2 is enriched in the promoters of genes associated with the TGF-β, Wnt, and cadherin signaling pathways. Wnt signaling plays an important role in both self-renewal and meso/endodermal lineage differentiation of hESCs.^23,24^ Similarly, cadherin signaling may play a role in the initial step of MSC differentiation, as cell–cell contacts and cellular migration are prerequisites for meso/endodermal differentiation.^25^ The TGF-β superfamily, which includes BMP4 and BMP2, has been implicated in stem cell differentiation towards mesenchymal lineages, particularly osteogenic lineages.^26,27^ Unexpectedly, when gene expression was individually assessed in MSCs and hESCs, GDF6 emerged as the most upregulated gene among the TGF-β superfamily members that were inversely associated with EZH2 enrichment. BMP4 and BMP2 were found to promote the differentiation of hESCs into mesoderm, and there have also been some studies showing that BMP4 may induce hESC differentiation into an extraembryonic lineage.^28^ GDF7, another upregulated factor in our analysis, was reported to be expressed in a more restricted pattern compared with that of GFD6 during mouse limb formation.^20^ GDF6/7 and ascorbic acid were reported to promote tenogenic differentiation of hESCs.^29^ In this study, we revealed that GDF6 promoted hESC differentiation into the MSC lineage. However, the function of GDF6 might not be restricted in mesodermal differentiation, as GDF6 has also been implicated in neural differentiation in eye and ear development.^30,31^ In addition to embryonic development, GDF6 was found to be associated with terminal MSC differentiation.^32,33^ However, although GDF6 plays an important role in the induction of hESC differentiation into mesoderm based on our studies, we do not know whether GDF6 directly regulates MSC differentiation during the mesodermal differentiation of hESCs.

We found that GDF6 was not expressed in hESCs, which was consistent with previous findings in induced pluripotent stem cells that showed that GDF6 expression was dramatically upregulated upon induction of differentiation.^31^ Compared to MSCs, hESCs showed greater enrichment of the repressive H3K27me3 modification the *GDF6* promoter. Although we did not find significant differences in enriched H3K4me3 levels at the *GDF6* promoter between hESCs and ES-MSCs, there might be other epigenetic mechanisms involved in the regulation of *GDF6* expression. Suppressor of zeste 12, another subunit of polycomb repressive complex 2, was also found to be present at *the GDF6* promoter, which is important for the maintenance of ESCs.^34^ Based on the genome sequencing of patients with nonsyndromic cochlear aplasia, *GDF6* expression was found to be controlled by *cis*-regulatory elements located within ~500 Kb pairs of the genome during ear development.^31^ Since GDF6 is required for the development of multiple tissues and organs, more efforts are needed to investigate the factors governing GDF6 expression in a temporal and spatial context.

Finally, when added to hESC culture, GDF6 selectively promoted the expression of mesodermal and MSC markers. Our results suggest that GDF6 plays an important role in the induction of hESC differentiation into mesoderm. More importantly, the purified MSCs derived from GDF6-treated hESCs exhibited osteogenic and chondrogenic differentiation potentials similar to those of the control cells, indicating promising clinical utilization for regenerative medicine. Together with our previous work, the present study provides strong evidence that GDF6 is a repressive target of EZH2 during hESC differentiation and that it can be utilized to enrich MSC production.

## Materials and methods

### ChIP-seq and RNA-seq data analyses

Raw ChIP-seq data for EZH2 in H1 hESCs were downloaded from the GEO database (GSE32509). Undifferentiated H1 hESCs were cultured in TeSR media on Matrigel (Cellular Dynamics).^35^ Raw ChIP-seq data for the H3K4me3 and H3K27me3 histone modifications and RNA-seq data of H1 hESCs and H1-derived hES-MSCs were downloaded from the NCBI epigenome roadmap, which was carried out by UCSD (http://www.ncbi.nlm.nih.gov/geo/roadmap/epigenomics/).^13,36^ Early passages of hES-MSCs generated from H1 hESCs were utilized for ChIP-seq and RNA-seq, and data analysis was performed as described previously.^16^

### Cell culture and MSC differentiation

All hESC experiments were conducted according to the protocol approved by the UCLA Embryonic stem cell research oversight committee (IRB: 10-001711-CR-00001). Both H1 and H9 hESCs (passages 35–45) grown on mitomycin C-treated mouse embryonic fibroblasts were acquired from the UCLA Broad Stem Cell Research Center and maintained as previously described.^16^ To rule out the possible effects of treatments on the feeder cells, hESC colonies were transferred onto matrigel-coated tissue culture dishes by using type IV collagenase (Cayman Chemical, Chemical, Ann Arbor, MI, USA) at a concentration of 1 mg·mL^−1^ and grown in mTeSR1 medium (STEMCELL Technologies, Vancouver, Canada). The cells were treated with GSK126 (CAS No. 1346574-57-9, Cayman Chemical), GDF6 (R&D Systems, Minneapolis, MN, USA) or vehicle control for 3 days as indicated. After the 3-day treatment, the cells were digested using trypsin (Invitrogen) to generate a single-cell suspension and further differentiated for 5 days on tissue culture dishes (Corning, NY, USA). Finally, the derived cells were either subject to induction for osteogenic and chondrogenic differentiation or sorted by flow cytometry.

### Characterization of osteogenic, chondrogenic, and adipogenic phenotypes

In all, 1 × 10^5^ derived cells were seeded per well in 12-well plates, and cells were induced to undergo osteogenesis, chondrogenesis, and adipogenesis as previously described.^37^^,^^38^ ALP activity assays and ARS staining were performed to evaluate ALP activity and extracellular matrix (ECM) mineralization, respectively, after OI.^37,38^ Alcian blue staining was performed to visualize cartilage deposition in the ECM after 3 weeks of chondrogenic induction. After 3 weeks of adipogenic induction, lipid droplet formation was detected with Oil Red O staining according to the manufacturer’s instructions (Diagnostic BioSystems, Pleasanton, CA, USA).

### Flow cytometry and fluorescence-activated cell sorting

To detect the expression of cell surface markers, the differentiated cells were collected and suspended in FACS buffer (phosphate-buffered saline (PBS), 10 mmol·L^−1^ EDTA, and 2% FBS) at a density of 2 × 10^6^ cells per ml. Cells were incubated with fluorescent-dye-conjugated antibodies for 30 min on ice in the dark. After being washed three times in FACS buffer, the cells were suspended in PBS. Sorting gates were established based on comparisons with isotype negative controls and compensation controls. Finally, the purified cells were collected into serum-free DMEM media for mRNA expression analysis or into complete media for cell propagation in vitro. The following conjugated antibodies (all from Biolegend) were used in this study: PerCP-CD45 (Cat No.: 368506), APC-CD73 (Cat No.: 344006), FITC-CD146 (Cat No.: 361012), and PE-CD271 (Cat No.: 345106) as previously described.^16^

### Quantitative reverse transcriptase-polymerase chain reaction (qRT-PCR)

An RNeasy Mini kit (Qiagen, Hilden, Germany) was used to isolate total RNA. First-strand cDNA was generated from aliquots of 0.5–2 μg of total RNA using random hexamers and reverse transcriptase according to the manufacturer’s instructions (New England Biolabs, Rowley, MA, USA). As previously described, qRT-PCR was performed using the QuantiTect SYBR Green PCR kit (Qiagen) on a CFX96 Touch™ Real-Time PCR Detection System (Bio-Rad, Hercules, CA, USA).^16^ Primers targeting following genes were used: glyceraldehyde 3-phosphate dehydrogenase (*GAPDH)*, 5′-GGA GCG AGA TCC CTC CAA AAT-3′ (forward) and 5′-GGC TGT TGT CAT ACT TCT CAT GG-3′ (reverse); *forkhead box F1 (FOXF1)*, 5′-CCC AGC ATG TGT GAC CGA AA-3′ (forward) and 5′-ATC ACG CAA GGC TTG ATG TCT-3′ (reverse); *msh homeobox 1 (MSX1)*, 5′-ACAC AAG ACG AAC CGT AAG CC-3′ (forward) and 5′-CAC ATG GGC CGT GTA GAG TC-3′ (reverse); *T-box transcription factor T (TBXT)*, 5′-TAT GAG CCT CGA ATC CAC ATA GT-3′ (forward) and 5′-CCT CGT TCT GAT AAG CAG TCA C-3′ (reverse); *kinase insert domain receptor (KDR)*, 5′-GGA ACC TCA CTA TCC GCA GAG T-3′ (forward) and 5′-CCA AGT TCG TCT TTT CCT GGG C-3′ (reverse); *GATA binding protein 4 (GATA4)*, 5′-CGA CAC CCC AAT CTC GAT ATG-3′ (forward) and 5′-GTT GCA CAG ATA GTG ACC CGT-3′ (reverse); *cluster of differentiation 73 (CD73)*, 5′-TTA CAC AGG CAA TCC ACC TTC-3′ (forward) and 5′-TTA CAC AGG CAA TCC ACC TTC-3′ (reverse); *CD146*, 5′-CTG CTG AGT GAA CCA CAG GA-3′ (forward) and 5′-CAC CTG GCC TGT CTC TTC TC-3′ (reverse); *CD271*, 5′-CCT CAT CCC TGT CTA TTG CTC C-3′ (forward) and 5′-GTT GGC TCC TTG CTT GTT CTG C-3′ (reverse); *alkaline phosphatase (ALPL)*, 5′-ACC ACC ACG AGA GTG AAC CA-3′ (forward) and 5′-CGT TGT CTG AGT ACC AGT CCC-3′ (reverse); *integrin binding sialoprotein (IBSP)*, 5′-CCC CAC CTT TTG GGA AAA CCA-3′ (forward) and 5′-TCC CCG TTC TCA CTT TCA TAG AT-3′ (reverse); *runt-related transcription factor 2 (RUNX2)*, 5′-GCA AGG TTC AAC GAT CTG AG-3′ (forward) and 5′-GGA GGA TTT GTG AAG ACG GT-3′ (reverse); *osteocalcin (OCN)*, 5′-GGC GCT ACC TGT ATC AAT GG-3′ (forward) and 5′-GTG GTC AGC CAA CTC GTC A-3′ (reverse); *SRY-box 9 (SOX9)*, 5′-AGG AAG CTC GCG GAC CAG TAC-3′ (forward) and 5′-GGT GGT CCT TCT TGT GCT GCA C-3′ (reverse); *collagen type II alpha 1 chain (COL2A1)*, 5′-CCT GGC AAA GAT GGT GAG ACA G-3′ (forward) and 5′-CCT GGT TTT CCA CCT TCA CCT G-3′ (reverse); *peroxisome proliferator-activated receptor gamma (PPARγ)*, 5′-TAC TGT CGG TTT CAG AAA TGC C-3′ (forward) and 5′-GTC AGC GGA CTC TGG ATT CAG-3′ (reverse); and lipoprotein lipase (*LPL)*, 5′-AGG ATG TGG CCC GGT TTA TC-3′ (forward) and 5′-CCA GGC TGT ATC CCA AGA GAT-3′ (reverse).

### Chromatin immunoprecipitation (ChIP)

ChIP assays were conducted using a ChIP assay kit according to the manufacturer’s instructions (Millipore). Briefly, cells were washed twice with PBS, crosslinked with 1% formaldehyde (Fisher) for 10 min at 37 °C, and then subjected to SDS lysis buffer containing 1 mmol·L^−1^ phenylmethylsulfonyl fluoride. The crosslinked DNA–protein complexes were sonicated and sheared into genomic DNA fragments ~200–500 bp in length. The genomic DNA fragments associated with proteins were immunoprecipitated with anti-human EZH2 antibodies or anti-human H3K27me3 antibodies (active motif). The precipitated complex was digested with proteinase K and purified using a ChIP DNA Clean & Concentrator kit (Zymo Research). The ChIP–DNA complexes were quantitatively measured using qPCR, and the data are shown as the percentage of input DNA. The primers for specific regions of the *GDF6 promoter* are as follows: forward, 5′-ATC TGC CTA AAT CGC TTT TAT CC-3′; reverse, 5′-CAA TAA TGT GGA AGG AGA CAG GA-3′. The primers for flanking *GDF6* are as follows: forward, 5′-GAT GTT GAA AGA AGC ACC AAC AC-3′; reverse, 5′-AAC AAG TTT TCA GGT GAT GCT GA-3′.

### Transplantation in nude mice

All animal procedures were performed as indicated in the protocol approved by the UCLA Animal Research Committee (ARC). Sorted CD73^+^CD146^+^CD271^+^CD45^−^ MSCs (1 × 10^6^) were incubated with 40 mg of hydroxyapatite/tricalcium phosphate scaffolds for 4 h at 37 °C and subcutaneously transplanted into 8-week-old nude mice (*n* = 5). The animals were sacrificed at eight weeks post transplantation. Samples were decalcified by 10% EDTA (pH = 7.4), sectioned, and stained with H&E for histological analysis. SPOT 4.0 software (Diagnostic Instruments, Sterling Heights, MI, USA) was used for quantification of the mineralized tissue in the H&E-stained samples.
